# Oil body membrane proteins as sensors of salt stress in sunflower seedlings: a proteomic insight

**DOI:** 10.1080/15592324.2025.2548313

**Published:** 2025-08-26

**Authors:** Mansi Gogna, Satish C. Bhatla

**Affiliations:** a Department of Botany, Maitreyi College, University of Delhi, Delhi, India; b Department of Botany, University of Delhi, Delhi, India

**Keywords:** Salt stress, sunflower, oil body membrane proteins, proteomics, oleosins

## Abstract

Sunflower, an important oil-yielding crop of tremendous economic importance worldwide, is sensitive to salt stress like many other agriculturally important crops. Different varieties of sunflower exhibit notable variations in their sensitivity/tolerance to salt-stress. Sensing of salt stress in sunflower is evident as early as at the seedling stage. Oil bodies, the major storehouse of fatty acids, are encased in a phospholipid monolayer containing intrinsic and extrinsic proteins. Any changes expected in the fatty acid composition of oil bodies as a response to salt stress are first perceived through alterations in the expression of oil body membrane proteins (OBMPs). The present investigations provide an in-depth proteomic analysis of OBMPs in the seedling cotyledons of three sunflower varieties exhibiting variations in their salt sensitivity. The exhaustive data from the LC‒MS/MS analysis of OBMPs highlight the differences in the levels of expression of a number of intrinsic and transiently expressed protein constituents of oil body membranes. The present proteomic analysis, thus, provides an insight into proteins capable of sensing salt stress as an early signaling response in sunflower seedlings.

## Introduction

Oil bodies (OBs) are dynamic entities present in oilseed plants, particularly in seeds, constituting 20%–50% of their overall mass, that are structurally attuned to play a crucial role in regulating the mobilization of neutral lipid store of triacylgycerides (TAGs).^1^^,^^2^ Within the phospholipid monolayer of OBs, several intrinsic proteins are embedded. These proteins principally include oleosins, caleosins, and steroleosins.^3^ These oil body membrane proteins (OBMPs), along with transiently expressed several other extrinsic proteins (such as proteases, lipases, lipoxygenases, etc.), play crucial roles in stabilizing and mobilizing the oil bodies in a precise and gradual manner depending on the phase of seedling growth under the prevailing environmental conditions of biotic and abiotic stress.^1^^,^^4^^,^^5^ Different isoforms of oleosins are likely to play different roles in maintaining the structural stability of oil bodies and lipid accumulation.^6^ The shape of OBs is commonly noted to vary from circular to ovoid with a size bandwidth of diameter from 0.5 to 2.5 µm.^2^^,^^7^ Three-dimensional reconstruction employed to decipher the seed volume of *Brassica napus* showed them to be less than 100 µm^3^.^8^ Proteomic investigation of oil body membrane proteins (OBMPs) has earlier been carried out in jatropha, mustard, sunflower, scutellum of maize, and diatoms.^9-14^ The most abundant protein present in the OB membrane has been cloned from maize and has been identified as an alkaline protein termed oleosin.^2^

One of the major functions of oleosins includes their ability to stabilize and resolve OBs during seed germination, desiccation, viability during winters, and lipid mobilization during seed germination.^2^ Oleosins facilitate the stabilization of OBs by inducing a uniform negative charge on the OB surface that limits their coalescence due to steric hindrance and electrical repulsion.^15^ The size of OBs is inversely proportional to the abundance of oleosins in the OB membrane.^4^^,^^16^ In Arabidopsis, oleosins not only influence the size of OB but also their spatial distribution. OB stabilization during seedling development in soybean, olive and *Pongamia* corresponds with their fusion.^17^ A transcriptomic approach demonstrated that oil body-associated protein 1 (OBAP1) is involved in the stability of oil bodies in the maize scutellum during seed maturation, and its expression decreases rapidly after germination.^12^

Identified as seed-specific proteins, **caleosins** possess stress-regulatory ability in diverse life forms.^18^ First reported as Sop1 from *Sesamum indicum* seeds, caleosins have the unique ability to bind calcium ions and oleosin-like proteins. Similar observations on caleosins have been made from fungi and single-celled algae, indicating their ancestral lineage.^2^^,^^19^ Additionally, caleosins serve as OB stabilizers. In cycads, artificial OBs and knockdown oleosin mutants of soybean, the absence of oleosins correlates with overaccumulation of caleosins, reflecting a compensatory mechanism to perpetuate OB’s structural stability.^20-23^ Contrary to the association with diverse life forms noted in oleosins and caleosins, steroleosin homologs isolated from the genomes of Arabidopsis and sesame show ancestral lineage association with mammals.^24^

Proteomic analysis of OB membrane proteins in the seeds of three varieties of *Jatropha curcas* revealed that the ratio of lipid content to caleosins abundance is involved in the regulation of OB size.^13^ Analyses of lipid droplets of *Sesamum indicum* indicated that some of the established marker proteins of the endoplasmic reticulum (disulfide-isomerase 2 precursor, calreticulin, and BiP) are also the components of lipid droplets.^10^ Based on these findings, oil bodies are no longer considered static storage organelles for neutral lipids and they seem to interact with various cellular organelles, such as glyoxysomes, peroxisomes, and mitochondria. The author’s laboratory earlier reported proteomic analysis of OBMPs at different stages of sunflower seed development whereby a change in the expression of different isoforms of oleosins, as well as transient migration of several other proteins from the cytosol to the OB membrane, is evident.^11^

The economic value of sunflower depends on the quality and content of oil stored in the oil bodies (OBs) located in the cells of cotyledons. In view of this, it is necessary to explore the components and functions of OBs through proteomic analysis. It is further important to understand the interaction of OB-associated proteins and their contributions to OB formation, stabilization, regulation of OB size, and lipid accumulation under harsh environmental conditions of growth. A synergy exists between membranous constituents, which are responsible for the resistance to coalescence offered by OBs.^15^^,^^25 ^From the author’s laboratory, recent work on salt-sensitive and -tolerant sunflower variants have revealed the intricate relationship between the retention of major OBMPs and OB mobilization under conditions of environmental stress.^4^ This may represent a possible new aspect of the biological functions of the OBs during the oil body mobilization. The present work provides a detailed GC‒MS analysis of OBMPs from three seedling cotyledons of three sunflower varieties differing in their degree of salt stress sensitivity. The data have been analyzed in the context with physiological work and other plants to draw logical conclusions on the mechanisms of salt stress tolerance in plants.

## Materials and methods

### Growth parameters

Seeds of three sunflower (*Helianthus annuus* L.) varieties, DRSH 1 (salt tolerant), KBSH 53 (semi-salt tolerant), and PSH 1962 (salt sensitive), procured from the Indian Institute of Oilseeds Research, Hyderabad (Telangana, India), were treated with a mild detergent (Teepol), followed by washing in tap water. The seed surface was sterilized using 0.005% mercuric chloride for 2 min followed by thorough washing with distilled water. After imbibing the seeds in double distilled water for 2 h, they were placed on pre-soaked and sterilized, moist germination paper layered on plastic trays. The seedlings were raised in the dark, maintained at 24°C–26°C, and irrigated with half-strength Hoagland nutrient medium (control). Salinity stress was provided in the form of 120 mM NaCl, augmented to half strength Hoagland medium.^26^^,^^27^

### Lipid extraction and GC–MS analysis of fatty acids (FAs)

Total lipids were extracted, resolved by thin layer chromatography (TLC) on silica gel, scrapped, and collected in glass vials.^4^ The samples were washed using *n*-hexane and acetone on an ultrasonicator. Methanolic-HCl was added to the washed lipid extracts, which were incubated at 90°C for 1 h in a water bath, followed by centrifugation at 2,000× g for 2 min. The supernatant was processed to create methyl esterification of FAs in the samples. Fatty acid methyl esters (FAMEs) were analyzed by GC–MS (Model: 5977A MSD coupled with 7890B GC series, Agilent Technologies, USA).^28^

### Oil body membrane proteins (OBMPs): extraction, washing, and SDS–PAGE analysis

Cotyledons (5 g FW for each sample) were ground to fine powder using pre-chilled pestle and mortar. After resuspending in the extraction buffer [0.1 M Tris, 0.4 M sucrose, 1 mM EDTA, 1 mM PMSF, and 0.2% *β*-mercaptoethanol] and vortexing for 30 min, the aliquots were centrifuged at 10,000× g for 20 min at 4°C, followed by sodium bicarbonate washing for the extraction of peripheral and integral membrane proteins.^29^ The OBMPs were separated and purified via several steps of bicarbonate washing protocol as already published.^30^The OBMP proteins were quantified in each sample using Markwell’s Reagent.^31^ Vertical SDS-PAGE (4**%**–15% gradient) was undertaken at a fixed current and gradient series of voltage: 25, 35, 55, and 75 for 10 min each, followed by at 100 V for 20 min and at 150 V till the dye front exits the gel, using MINI-PROTEAN Cell units (Bio-Rad, USA). The gel was then stained using 0.3% solution of Commassie Brilliant Blue (R−0). Destaining of the gel was carried out using the destaining solution (water:methanol:acetic acid in the ratio of 50:40:10).

### Detection of aquaporin isoforms by western blot analysis

After successful extraction of OBMPs by bicarbonate washing,^30^ aliquots of OBs containing 75 µg proteins were separated on a pre-cast gradient linear gel (4%–15%) (Bio-Rad, USA). After gel electrophoresis as mentioned above, the gel was removed from the casting unit and washed with transfer buffer (25 mM Tris containing 192 mM glycine, 0.1% SDS, and 20% methanol). The nitrocellulose (NC) membrane was activated in double distilled water for 10 min, followed by incubation in transfer buffer for 15 min. The blot was incubated with blocking buffer (7% BSA, 0.02% Tween−0 dissolved in phosphate-buffered saline, pH 7.4) for 2 h at room temperature to prevent any non-specific binding. This was followed by the addition of primary antibody (dilution 1:1000) against the respective aquaporin’s isoform: TIP1 & 2 and PIP1 & 2 (Thermo Fisher Scientific, USA) and an overnight incubation at 4°C. After thorough washing with wash buffer (0.2% Tween in PBS), the blot was incubated with secondary anti-body (IgG, alkaline phosphatase raised in goat, 1:2000) for 2 h, followed by washing with wash buffer for 15 min. A fresh solution of NBT/BCIP (1 tablet of nitro blue tetrazolium chloride/5-bromo−4-chloro−3 indolyl phosphate, toluidine salt dissolved in double distilled water) solution was used to visualize the aquaporin isoforms.

All the experiments were performed in triplicates.

### Proteomic analyses of oil body membrane proteins (OBMPs)

**Figure 1.**
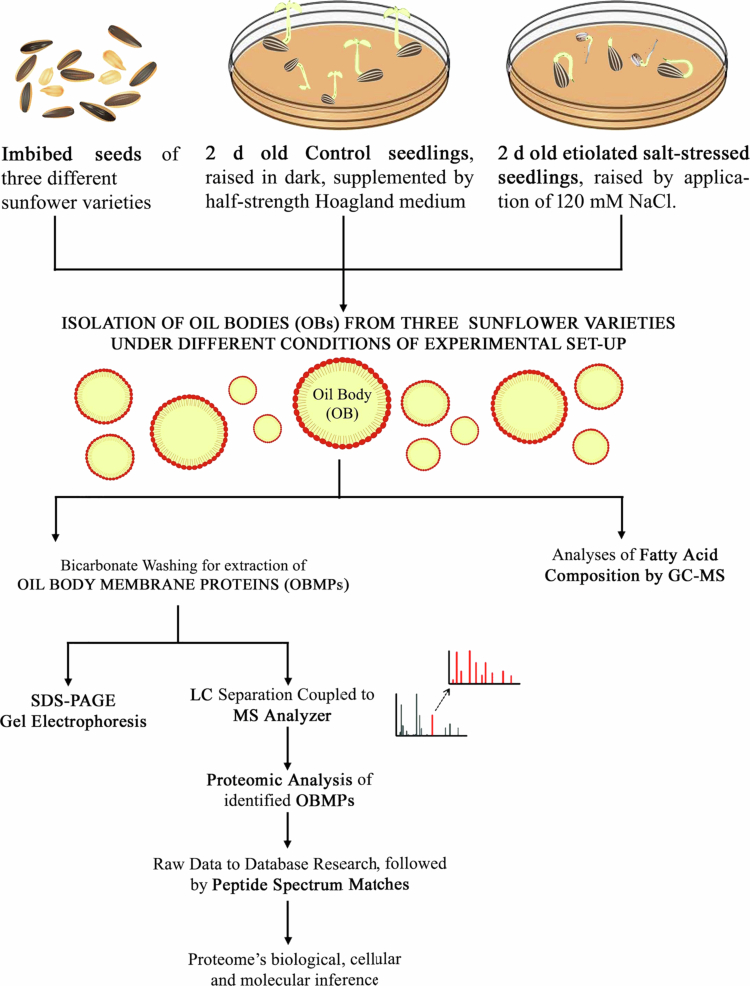
. Flow chart depicting an overview of methodology followed for research conducted on oil body membrane proteins (OBMPs) isolated from three varieties of *Helianthus annuus* L. under three conditions, namely imbibed seeds, dark grown seedling cotyledons of seedlings raised in the presence, and absence of 120 mM NaCl

Extracted aliquots of OBMPS were processed to prepare desalted tryptic peptides,^32^ followed by LC–MS/MS analysis on an LTQ-Orbitrap XL (Thermo Fisher Scientific, USA). The prepared samples were transferred to a trap column. After a 5 min run, peptides were circumvented and separated on an analytical C18 reversed-phase resin column by reversed phase chromatography operated on a nano-HPLC system (Ultimate 3000, Dionex) along with a non-linear gradient, using 5% acetonitrile in 0.1% formic acid solution in water (A) and 0.1% formic acid in 98% (B) at a flow rate of 300 nl min^−1^. The gradient settings were operated as follows: 5–140 min: 14.5–90% A–95% B, 145–150 min: 95% B, followed by equilibrium for 15 min to initial conditions. From the MS prescan, the 10−5 most abundant peptide ions were selected for fragmentation in a linear trap if they transcended an intensity of at least 200 counts and had a double charge. For peptide identification, the Swiss–Prot database was used and disposed using Scaffold software.^32^ A flowchart depicting the materials and methods has been provided (Figure 1).

### Parameters of OBMP’s proteome data analysis

Data analysis was carried out by comparing the peptide spectrum matches (PSMs), unique peptide scores, sum posterior error probability (PEP) score and, finally, the abundance ratio (log 2) values, signifying the relative abundance of the identified proteins under stress with respect to those in the imbibed seeds and control (−NaCl) seedling samples, respectively. −Log 10 of *p*-values have been assessed to present significance of occurrence and abundance ratio of proteins with respect to their relative control. *p*-Values < 0.05 are indicative of significance whilst *p*-​​​​​values > 0.05 indicate that the proteins are insignificant, in terms of relative abundance and/or occurrence.

**Figure 2.**
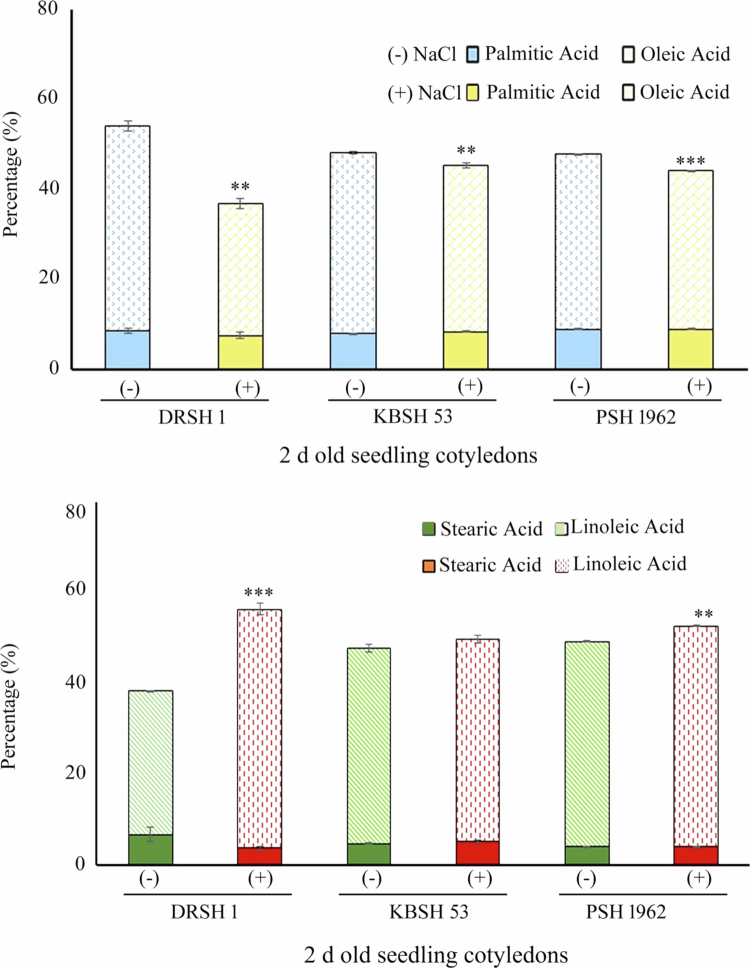
Profiling of major fatty acid composition of oleic, linoleic, stearic, and palmitic acids in 2 d old, dark-grown seedling cotyledons of three sunflower varieties, under the influence of salt stress. Statistical analysis was carried out using SPSS 22 (*p < 0.05, **p < 0.01, and ***p < 0.001) in comparison to control (without NaCl), according to one-way ANOVA. All the above data represent mean of three technical replicates drawn every time from three sets of biological experiments. Vertical bars represent “standard error.”

*The proteome finger print analysis* has been undertaken in two ways: 1. Comparative analysis of relative abundance ratios of OBMPs extracted from cotyledons of 2 d old control seedlings and salt-stressed 2 d old seedlings, *using imbibed seeds as control.* 2. Comparative analysis of relative abundance ratio of OBMPs was undertaken from salt stressed 2 d old seedlings (+NaCl), using imbibed seeds as well as 2 d old control (−NaCl) seedling cotyledons as controls, respectively. The abundance ratio of OBMPs extracted from the seedling cotyledons raised in the absence or presence of 120 mM NaCl was analyzed using data from OBMPs extracted from imbibed seeds as control. This highlights their up- or downregulation with respect to the control (imbibed seeds). Some membrane proteins do not show any significant regulation with respect to imbibed seeds. Hence, they have been accounted as proteins showing common expression or with insignificant differences. (−NaCl) depicts the abundance ratio of OBMPs extracted from 2 d old control seedling cotyledons relative to the OBMP expression of imbibed seeds. Thus, the unique positive expression of OBMPs in control (−NaCl) signifies the unique upregulation or expression of proteins in 2 d old seedling cotyledons (−NaCl) relative to OBMPs obtained from imbibed seeds, while unique negative expression/downregulation of OBMPs signifies their presence in control (imbibed seeds). The term “120 mM NaCl” depicts the abundance ratio of OB membrane proteins identified from 2 d old salt (120 mM NaCl)-stressed seedling cotyledons relative to the OBMP expression of imbibed seeds. Thus, unique expression in salt-stressed seedling cotyledons signifies the expression of unique OBMPs relative to the OBMPs obtained from imbibed seeds (control). The data on the distribution of OB membrane proteins extracted from the seedling cotyledons of three sunflower varieties were also analyzed on the basis of *Log 2 value of abundance ratio*. “+NaCl/imbibed or 120/imbibed” depicts the unique expression of OB membrane proteins in comparison to the OB membrane proteins extracted from imbibed seeds. “120/0” highlights the unique expression of OB membrane proteins in comparison to the OB membrane proteins extracted from 2 d old control seedling cotyledons. Comparison of the proteome of salt-stressed seedling cotyledons with the one extracted from control (−NaCl) seedling cotyledons showed proteins which exhibit unique expression in response to salt, while abundance ratio relative to imbibed seeds reveals response to the seedling’s temporal and physiological changes from imbibed seeds to 2 d old seedlings. *Volcano plot/Scatter-plot* was also generated using two independent variables, i.e., the abundance ratio (log 2-fold change) and log 10 (*p*-value). The cartesian coordinates display values on the scatter-plot are depicted using different colors. The data are displayed as a collection of points, each having the value of the abundance ratio (log 2-fold change) on the horizontal axis (*x*-axis), while the log 10 value (*p*-value) determines the position on the vertical axis (*y*-axis). It signifies a correlation between the abundance ratios and *p*-value of the samples analyzed. Using volcano plots, the relation and trend of three sets were revealed (Figure 2).

## Results and discussion

### Salt sensitivity/tolerance of sunflower seedlings is reflected in their early growth response

**Figure 3.**
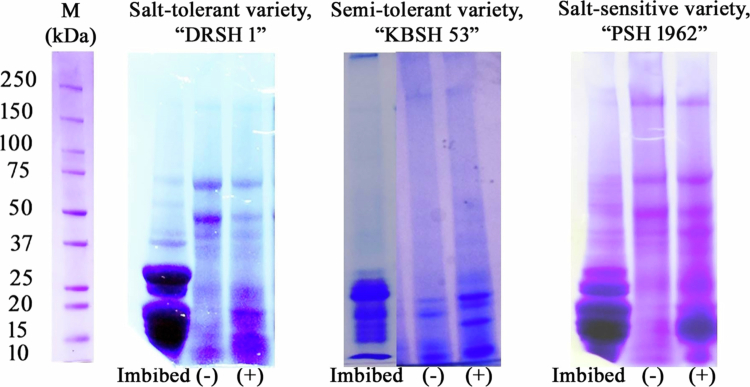
Oil body (OB) membrane protein mobilization as affected by NaCl (120 mM) treatment. Oil bodies were extracted from imbibed and 2 d old seedling cotyledons, grown in dark, subjected to bicarbonate washing, and resolved on a 4%–15% gradient gel. Delayed OB mobilization (longer retention) is observed under salt stress in salt-tolerant (DRSH 1), semi-tolerant (KBSH 53), and sensitive varieties (PSH 1962) of sunflower. (−) indicates absence of NaCl, whereas (+) indicates treatment with 120 mM NaCl.

During the early phase of seedling growth in the three varieties of sunflower, maintenance of the apical hook and extension growth of the primary root did not exhibit any differences in normal (unstressed) growth conditions. Apical hook formation, however, gets abolished in response to salt stress (120 mM NaCl) in all three varieties. Primary root extension is also significantly reduced in semi-salt-tolerant and salt-sensitive varieties but it remains unaffected in salt-tolerant varieties in response to salt stress. The impact of salinity stress on the early phase of seedling growth is also evident in the fresh weight of the cotyledons in the three sunflower varieties (Table 1). While the mean fresh weight of cotyledons is highest in the imbibed seeds of salt-sensitive variety (PSH 1962), the impact of salt stress on the decline in the fresh weight of cotyledons of their 2 d old seedlings is also strongest as compared to that in the salt-tolerant (DRSH 1) and semi-salt-tolerant (KBSH 53) varieties under investigation. In congruence with these observations, a reduction in plant height, dry weight, and relative growth rate have also been earlier been reported in salt-sensitive and salt-tolerant genotypes of rice.^33^ Likewise, a decline in growth and crop productivity has also been reported in wheat under salt stress.^34-36^ However, in seedlings of *Eruca sativa* (an edible vegetable crop plant of the family Brassicaceae), no significant deviation in fresh weight is noted under salinity stress below 137 mM NaCl.^37^ It is thus evident that the impact of salt stress on plant growth and development is evident within 48 h of application, i.e., at the seedling stage (Figure 3).

**Table 1.** Impact of salt stress on the fresh weight of cotyledons derived from dark-grown seedlings of salt-tolerant, semi-salt-tolerant, and salt-sensitive varieties of sunflower.

Fresh weight (g) (FW)	DRSH 1 (salt-tolerant variety)			KBSH 53 (salt semi-tolerant variety)			PSH 1962 (salt-sensitive variety)		
	IS	(−)	(+)	IS	(−)	(+)	IS	(−)	(+)
Average + SE	0.067 ± 0.0002	0.056 ± 0.0017	0.028 ± 0.0014**	0.063 ± 0.0014	0.059 ± 0.0016	0.022 ± 0.001*	0.079 ± 0.001	0.045 ± 0.0019	0.015 ± 0.0016*

*and **, indicate the statistical significance with *p*-value > 0.05, 0.01, respectively. IS: imbibed seeds. (−): 2 d old seedlings raised under control conditions; (+): 2 d old seedlings raised in the presence of 120 mM NaCl. Each fresh weight value is a mean of 30 cotyledons.

### Oil bodies are differently affected in the three sunflower varieties in response to salt stress in terms of fatty acid composition and mobilization of oil body membrane proteins (OBMPs)

**Figure 4.**
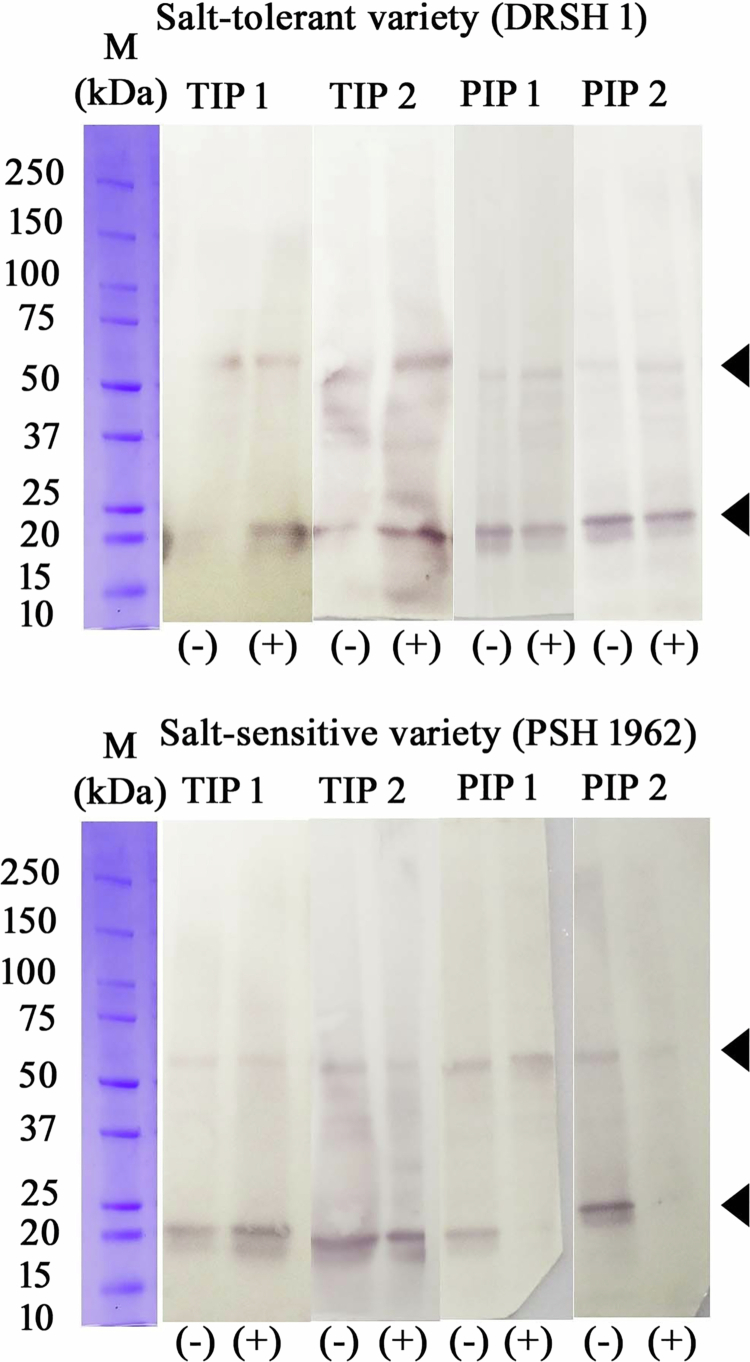
Expression of aquaporins in OB membrane proteins extracted from the OB fraction in the absence and presence of 120 mM NaCl. Western blot analysis of the effects of aquaporins (TIP1, TIP2, PIP1, and PIP2) on bicarbonate-washed OBMPs isolated from seedlings of salt-tolerant (DRSH 1) and salt-sensitive (PSH 1962) varieties raised in the absence and presence of salt. (+) Indicates 120 mM NaCl. Noteworthy differences in the expression of TIP1 & 2 and PIP1 & 2 are evident in both varieties under stress. The experiments were repeated at least thrice.

Significant qualitative and quantitative differences have been observed in the fatty acid profiles of imbibed seeds and 2 d old seedling cotyledons by GC‒MS analyses.^4^ A noteworthy decline in the percentage of oleic and palmitic acids is evident, along with a surge in the content of stearic and linoleic acids in salt-tolerant (DRSH1) and salt-sensitive (KBSH 53) varieties in response to salt stress. The contents of major saturated fatty acids have previously been reported to undergo a sudden decline in response to salinity stress as an adaptative response in sunflower, mustard and maize^4^^,^^38^^,^^39^ (Figure 4).

A rapid mobilization of OBs occurs within 48 h of germination (2 d old seedlings) both in salt-tolerant and salt-sensitive varieties under control (−NaCl) conditions of growth, whereas in semi-salt-tolerant variety, OB mobilization is not evident. Salt stress leads to the retention of OBs in salt-sensitive variety though in salt-tolerant variety the impact of salt stress on OB mobilization is relatively much less. Interestingly, OB mobilization was lowest in the semi-salt-tolerant variety, irrespective of salt stress. Similar earlier investigations on OBs extracted from the leaves and seeds of *Persea americana, Arabidopsis thaliana, Hevea brasiliensis, Glycine max,* and *Oryza sativa* have reported that OB membrane proteins such as oleosins, caleosins, and steroleosins play crucial roles in inducing tolerance during seed germination by fending off homotypic fusion of oil bodies in cotyledons, resulting in longer retention of smaller-sized OBs under stressful conditions.^7^^,^^23^^,^^40-44^ Oleosins, in particular, are known to play a prominent role in imparting steric hindrance and electronegative repulsion for the stability of oil body.^6^^,^^11^^,^^45^

**Figure 5.**
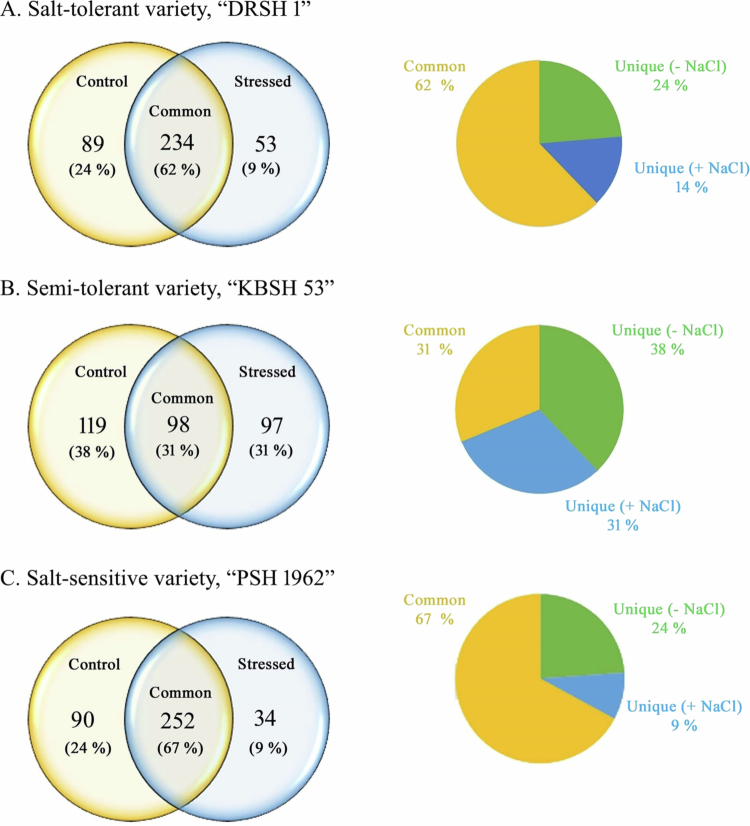
Comparative analyses of OB membrane proteins extracted from cotyledons of 2 d old seedlings of A. Salt-tolerant variety, “DRSH 1.” B. Semi-tolerant variety, “KBSH 53.” C. Salt-sensitive variety, “PSH 1962,” using imbibed seeds used as a control. Venn diagram exhibits unique and common expression oil body membrane proteins, whereas pie-chart depicts the overall distribution of total proteins.

Any evidence of oil body mobilization is expected to be substantiated with alterations of OB membrane proteins. SDS‒PAGE analysis of OB membrane proteins from the three varieties under investigation has shown similar profiles in imbibed seeds, but their mobilization within 48 h of seedling growth demonstrates noteworthy differences under control (−NaCl). Salt stress appears to facilitate a better retention of OB membrane proteins in all the three varieties (Figure 5). The clear impact of salt stress on OB mobilization by the retention of OB membrane proteins differentially in salt-sensitive, semi-salt-sensitive, and salt-tolerant varieties is evident as early as within 48 h of seed germination. The development of OBs is essential for the successful germination of seeds. Oleosins are directly involved in the size regulation of OBs. Abundant retention of oleosins on the OB membrane surface limits the fusion of OBs with one another, which is commonly noted under conditions of distress. Additionally, the calcium-binding protein, caleosin, has also been observed in some oil-yielding crops in close association with stress-responsive pathways.^7^ To further corroborate these observations, expression of steroleosins, namely A and B, extracted and purified from seeds of *Arabidopsis* and *Helianthus annuus* L., has shown aggravated retention under conditions of stress.^46^^,^^47^ These observations form the basis of a thorough proteomic analysis of OBMPs to decipher early events of signaling response due to stress.

### Impact of salt stress on the proteome profiling of OBMPs in the imbibed seeds and seedlings

**Figure 6.**
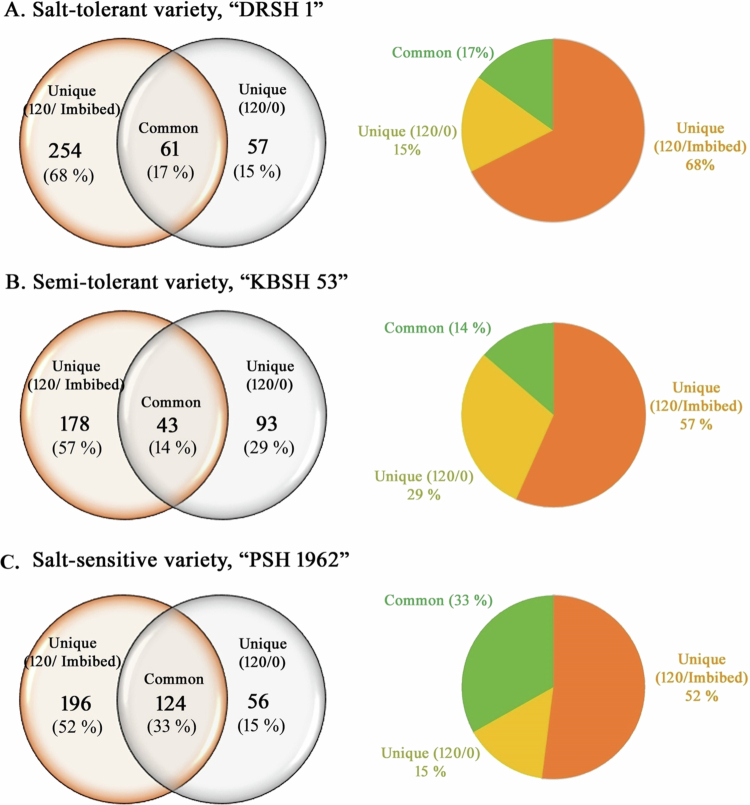
Comparative analyses of the abundance ratio of OB membrane proteins extracted from 2 d old treated (120 mM NaCl) seedling cotyledons of A. salt-tolerant, “DRSH 1” variety, B. semi-tolerant variety, “KBSH 53,” and C. salt-sensitive variety, “PSH 1862” relative to OBMP extracts isolated from imbibed seeds (120/imbibed) and 2 d control seedling cotyledons (120/0). The Venn diagrams depict the unique expression of various oil body membrane proteins in comparison to the protein extracts from 2 d old control seedling cotyledons. The effects of salt stress (120 mM NaCl) on 57 and 56 oil body membrane proteins (OBMPs) were evident in the salt-tolerant and salt-sensitive varieties, respectively. The pie-chart representations reveal unique expression of OBMP from salt-stressed seedlings.

**Figure 7.**
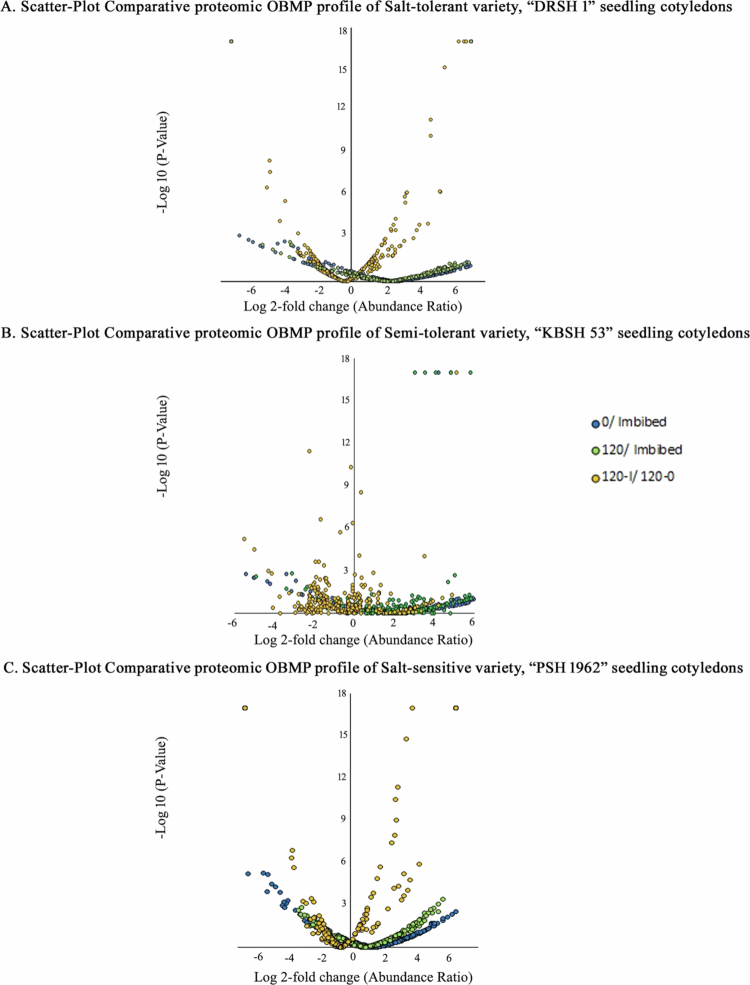
Scatter-plot reveals the correlation between abundance ratios and *p*-values of the samples. A, B, and C show the correlations, similarities, and differences in the distribution of membrane proteins extracted from three samples extracted from seedling cotyledons of the salt-tolerant variety (DRSH 1), semi-tolerant variety (KBSH 53), and salt-sensitive variety (PSH 1962), respectively.

**Figure 8.**
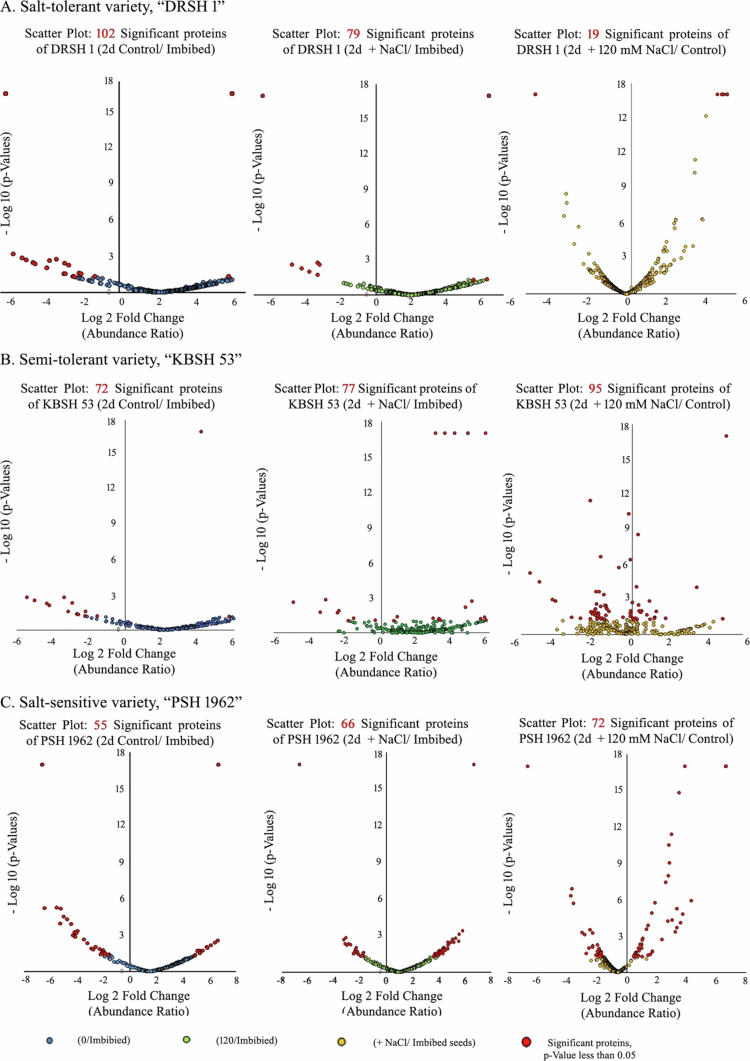
. Volcano plot representation of proteome profile on the basis of log 2-fold change of the abundance ratio and −log 10 of the *p-*values, less than 0.05. The higher the log 10 (*p*-value) is, the more statistically significant the data are. Significant proteins with *p-*values less than 0.05 are highlighted in red across all three volcano plots. Various proteome profiles from three sunflower varieties exhibiting significant modulations with respect to their controls were noted in 2 d control (−) NaCl)/imbibed seeds, 2 d stressed (+) NaCl/imbibed seeds, and 120 mM NaCl/(−) NaCl, respectively

***Salt-tolerant sunflower variety (DRSH 1):*** The proteome profile of semi-salt-tolerant (KBSH 53), salt-tolerant (DRSH 1), and salt-sensitive (PSH 1962) has revealed significant differences in distribution of proteins in the isolated oil bodies. These proteins can be grouped into ten categories, namely primary metabolic, proteolytic, secondary metabolic, regulatory, signal transduction-associated, storage, stress response-related, structural, transport, and miscellaneous proteins (Tables 2–4). A western blot for the detection of aquaporins was carried out using antibodies specific to aquaporin isoforms—PIP1 and 2; TIP1 and 2 for the salt-tolerant (DRSH 1) and salt-sensitive varieties (PSH 1962). According to the work published from author’s laboratory, in the semi-tolerant variety, KBSH 53, the presence of aquaporins isoform TIP2 has been noted, downregulated in the presence of 120 mM NaCl in 2 d old cotyledons55. The results of the present study indicate that in the salt-tolerant (DRSH 1) and salt-sensitive varieties (PSH 1962), a remarkable upregulation in the abundance of TIP1 and TIP2 is evident under salt stress. In salt-sensitive variety, noteworthy decline in the abundance of aquaporins PIP1 and 2 has been recorded (Figures 6 and 7). A total of 2644 proteins were identified from the OB homogenates of the salt-tolerant variety (DRSH 1). Out of these, 376 proteins exhibit significant regulation by salt stress based on significant variations in their abundance ratio ( Table 2). The highest modulation by salt stress-induced modulation is evident in regulatory proteins (116), followed by transport proteins (77). Among storage proteins, the lowest number of proteins (9) exhibit regulation due to salt stress among storage proteins. The total number of stress-related proteins has been found to be higher in salt-tolerant variety (37), compared to salt-sensitive variety (25). Thirty-one percent of the isolated OBMPs from DRSH 1 are regulatory proteins, followed by (in decreasing order) transport proteins (20%), proteins associated with primary metabolism (11%), and proteins involved in the stress response (10%) (Table 2). Common OBMPs in the salt-tolerant variety (DRSH1) (234 proteins; 62%) show no significant shift in their expression relative to the control (imbibed seeds) (Figure 8A). Unique expression of proteins was noted in 2 d old seedlings raised under control as well as under salt stress. Nearly 14% of the proteins exhibit a unique pattern of expression under salt stress relative to the imbibed seeds of the salt-tolerant variety DRSH 1. Similarly, OBMPs extracted from salt-stressed seedling cotyledons have been analyzed both in comparison to the OBMPs extracted from imbibed seeds and control seedling cotyledons, which are used as controls (Table 2; Figure 5C).

**Table 2.** Major classes of oil body membrane proteins (OBMPs) extracted from oil body (OB) membrane fractions of imbibed seeds and seedling (2 d old) cotyledons of salt-tolerant variety of sunflower (DRSH 1).

Classes of proteins in sunflower varieties exhibiting regulation	DRSH 1 (salt-tolerant variety)	KBSH 53 (salt semi-tolerant variety)	PSH 1962 (salt-sensitive variety)
Primary metabolism	41	52	66
Proteolytic proteins	25	16	25
Regulatory proteins	116	65	146
Secondary proteins	20	–	10
Signal transduction proteins	25	04	17
Storage proteins	09	17	07
Stress response proteins	37	67	25
Structural proteins	20	39	21
Transport proteins	77	39	53
Miscellaneous proteins	06	16	06
**Total protein count**	**376**	**314**	**376**

Basis: LC‒MS/MS analysis.

**Table 3.** Regulation of expression pattern of OBMPs from 2 d, dark-grown, salt tolerant (DRSH 1), semi-tolerant (KBSH 53), and salt sensitive (PSH 1962) seedling cotyledons (control and salt stressed). Control: imbibed seeds.

Number of proteins expressed in OBMPs	DRSH 1 (salt-tolerant variety)		KBSH 53 (semi-salt-tolerant variety)	PSH 1962 (salt-sensitive variety)
Control seedlings (−NaCl)	89	119	196
Salt stressed seedlings (120 mM NaCl)	53	97	56
Common proteins (no significant difference in relative abundance)	234	98	124

Basis: LC‒MS/MS analysis.

**Table 4.** Impact of salt stress on OBMPs of seedling cotyledons. Controls: imbibed seeds and 2 d old seedling cotyledons, respectively.

Number of proteins expressed in OBMPs	DRSH 1 (salt tolerant)	KBSH 53 (salt semi tolerant)	PSH 1962 (salt sensitive)
Unique expression of proteins (+NaCl vs imbibed seeds)	254	178	196
Unique expression of proteins (120 mM NaCl vs –NaCl)	61	98	56
Common proteins (no significant difference in relative abundance)	61	43	124

Basis: LC‒MS/MS analysis.

While not much difference has been noted between control and stressed seedlings, compared with imbibed seeds as controls, salt-stressed seedlings exhibit notable deviations in their unique expression patterns (Figure 6). Scatter-plot correlation comparison revealed a remarkable deviation in the expression, abundance pattern, and *p*-values of protein expression in salt-stressed seedling cotyledons with respect to 2 d old control seedlings as control (Figure 7). A total of 102 proteins from OBMPs of seedlings raised in the absence of NaCl exhibit noteworthy response (Figure 8A). Various proteins exhibit significantly high upregulation. These include malate synthase, putative allene oxide synthase, putative DNAJ- a heat shock family protein, ribosomal protein L16, putative clathrin adaptor medium subunit family protein, acetyltransferase component of pyruvate dehydrogenase complex, putative ATP synthase subunit gamma, putative calcium-dependent protein kinase, uridine kinase, putative aquaporin PIP2−5, putative staurosporin, and temperature sensitive 3-like A, putative acetyl-CoA carboxylase, alpha subunit, transmembrane 9 superfamily member, multifunctional fusion protein, dirigent protein, putative fatty oxidation complex, alpha subunit FadB, putative molybdate-anion transporter, major facilitator superfamily domain protein, zeta-carotene desaturase, putative multidrug resistance-associated protein 2, putative GRIM-9, and protein Ycf2). A relatively less proportion of proteins were downregulated in the salt-tolerant variety raised in control (−NaCl) conditions. These include phosphoribulokinase, putative bifunctional kinase pyrophosphorylase, putative RuBisCO large subunit-binding protein subunit alpha protein, germin-like protein, putative ABC transporter F family member 2, putative seipin family, putative plastid transcriptionally active 17, and malic enzyme.

Similarly, in the cotyledons of the salt-tolerant variety (DRSH 1) raised in the presence of 120 mM NaCl, a fairly distinct proteome profile has been noted upon comparison with similar data from imbibed seeds as control. Unique expression of 79 proteins was evident. Proteins which exhibit high upregulation include putative DNAJ, a heat shock family protein, putative 50S ribosomal protein L12 protein, putative translocase of the inner mitochondrial membrane, putative clathrin adaptor complexes medium subunit family protein, ribulose bisphosphate carboxylase small chain, putative NAD(*P*)H dehydrogenase B2, acetyltransferase component of pyruvate dehydrogenase complex, putative 2-oxoglutarate dehydrogenase E1 component, putative ATP synthase subunit gamma, putative ATP synthase subunit delta, uridine kinase, putative calcium-dependent protein kinase, putative aquaporin PIP2−5, putative staurosporin and temperature sensitive 3-like A, protein Ycf2, putative GRIM−9, and many more.

Only 17 proteins are downregulated out of the listed 79, indicating a strong physiological defense mechanism employed by DRSH 1 to ameliorate salinity stress. Proteins such as the putative peptidase S8, subtilisin-related protein, tubulin gamma chain, putative transcription regulator, germin-like protein, seed storage albumin 4, and 11S globulin (Fragment) were downregulated. A comparison of the proteome profile extracted from the salt-stressed seedling cotyledons with the OBMPs of the control seedlings revealed that 19 proteins have been found to be significantly regulated by salinity. Of these, 12 were upregulated, whereas the remaining were significantly downregulated.

***Semi-salt-tolerant variety (KBSH 53):*** A total of 1386 proteins were identified by LC–MS analysis of the OB fractions. A total of 314 proteins exhibiting significant regulation due to salt stress have been identified. Salt stress results in the highest level of regulation among stress response proteins (67), followed by regulatory proteins (65) and proteins related to primary metabolism (40). Least regulation is evident among signal transduction proteins (04). Regulation of stress-related proteins was highest in the seedlings of semi-tolerant variety (67), followed by those of the salt-tolerant variety (37), and the salt-sensitive variety (25), in decreasing order. Detailed analysis of proteome profiles revealed that out of 314 oil body membrane proteins (OBMPs) shortlisted on the basis of significant variations in abundance ratios, 21% are regulatory proteins and stress response proteins (Table 2). The salt-sensitive variety (PSH 1962) exhibits the regulation of 146 regulatory and 25 stress response proteins, compared to 65 regulatory and 66 stress response proteins in semi-tolerant variety (KBSH 53). A total of 116 regulatory and 37 stress response proteins are regulated in the proteome of oil bodies extracted from 2 d old salt-tolerant (DRSH 1) seedlings. Details of the entire proteome isolated from cotyledons of semi-tolerant variety (KSBH 53) indicate no significant shift in the expression of common OBMPs (98 proteins; 31%) relative to the control (imbibed seeds) (Table 3; Figure 5B). Unique expression of proteins was noted in 2 d old seedlings raised under control as well as stressed conditions. Nearly 31% of the proteins exhibit a unique pattern of expression under salt stress relative to the imbibed seeds of semi-tolerant variety, KBSH 53 (Figure 5B). Similarly, the unique expression of 28% of the proteins (93 OB MPs) was noted under the influence of salinity stress (Figure 6B). Using volcano plots, the relationship and trend of the three sets were revealed. Noteworthy differences were noted between the control and stressed seedlings, against imbibed seeds used as a control. Similarly, salt-stressed seedlings exhibit remarkable deviations in the expression and abundance patterns of OBMPs with respect to control seedlings (Figure 7).

A total of 72 proteins from OB MPs of seedlings raised in the absence of NaCl exhibited noteworthy responses (Figure 8B). Various proteins which exhibit significant upregulation in 2 d old control (unstressed) seedlings following imbibition include chloroplastic ATP synthase subunit alpha, ATP synthase subunit beta, citrate synthase, ACT domain-containing small subunit of acetolactate synthase protein, phosphoglycerate kinase, peroxidase, ubiquitin, putative carrier protein, 26S proteasome non-ATPase regulatory subunit 1 homolog, peptidyl-prolyl cis-trans isomerase, histone H4, putative enolase 1, seed storage albumin 1, putative 11-S seed storage protein, plant RmlC-like jelly roll fold protein, putative universal stress protein A, putative heat shock protein 70 family, putative ADP, ATP carrier protein and putative lipase/lipoxygenase, and PLAT/LH2 family protein. Similarly, major proteins exhibiting downregulation in their abundance ratio include putative chaperone protein dnaK2, peptidylprolyl isomerase and the putative 14-3-3 domain-containing protein oleosin, and the putative dehydrin. A slight downregulation in the expression pattern was noted for elongation factor 1-alpha, putative glucose/ribitol dehydrogenase, oleosin, putative sterol carrier protein 2, putative aspartic peptidase A1 family, putative vacuolar ATP synthase subunit E1, and putative dehydrin.

A fairly distinct profile was evident in salt-stressed seedlings compared with imbibed seeds as control. Unique expression of 77 proteins was observed. Salt stress leads to upregulation of quite a few proteins (compared to imbibed seeds). These include putative vacuolar ATP synthase subunit E1, putative 40S ribosomal protein S5, putative seed maturation protein, putative leucine-rich repeat (LRR) family protein, putative succinyl transferase, putative major intrinsic protein, aquaporin-like protein, putative calnexin, Cu/Zn superoxide dismutase (fragment), Cu/Zn superoxide dismutase (fragment), seed storage albumin 2, 11S globulin (fragment), putative glutelin type-A 2 and B−1, putative legumin A and B, putative universal stress protein A, tubulin beta and putative lipase/lipoxygenase, PLAT/LH2 family protein, and oleosin. Downregulation is evident in the expression of glutamine synthetase, putative allene oxide synthase, putative peroxygenase, heat shock protein 17, putative V-type proton ATPase catalytic subunit A, putative embryonic protein DC-8, 14-3-3-like protein, and putative triacylglycerol lipase-like 1.

Regulation in the expression of 95 OBMPs is evident in salt-stressed seedling cotyledons in comparison to 2 d old control seedling cotyledons. Significant upregulation and *p*-value (> 0.001) are noted in the expression of proteins, such as putative RuBisCO large subunit-binding protein subunit alpha protein, putative cytochrome P450, AAI domain-containing protein, putative enolase 1, putative calmodulin, catalase, oleosin, 60S ribosomal protein L13, L23s, and L24, putative lipase/lipoxygenase, PLAT/LH2 family protein, and putative embryonic protein DC−8. Downregulation is evident in the expression of proteins such as malate synthase, putative triacylglycerol lipase-like 1, putative chaperone protein htpG family protein, putative regulatory particle triple-A ATPase 5A, tubulin alpha chain, and putative CHCH.

*** Salt-sensitive variety (PSH 1962):*** A total of *5244* proteins has been identified by LC‒MS analysis of the homogenates. A detailed proteome profiling and distribution analyses showed that out of the 376 oil body membrane proteins (OB MPs) shortlisted on the basis of significant differences observed in abundance ratios, 39% are regulatory proteins, followed by proteins associated with primary metabolism (17%), transport (14%), proteolytic and stress-response related (7% each), structural (5%), signal transduction (4%), secondary metabolism (3%), and storage proteins (2%) ( Table 2). The highest number of proteins modulated by salt stress belong to the categories of regulatory proteins (146). Least modulation (7 proteins) is evident in the case of storage proteins. Common OBMPs (252 proteins; 67%) show no significant shift in their expression relative to the control (imbibed seeds) (Table 3; Figure 5C). Similarly, OBMPs extracted from salt-stressed seedling cotyledons were analyzed, both in comparison to the OB membrane proteins extracted from imbibed seeds and control seedling cotyledons. A unique expression of 15% proteins is noted under NaCl stress, in comparison to OBMPs of (−NaCl) seedling cotyledons as control (Table 4; Figure 6C).

Volcano plot representation emphasized the stark correlation between the abundance ratio pattern and *p*-values of proteome profile in salt sensitive, PSH 1962 (Figure 7C). A total of 55 proteins from OBMPs of 2 d old control seedling cotyledons exhibit significant changes in their expression (Figure 8C). Various proteins which exhibit upregulation with high *p*-values include ribulose bisphosphate carboxylase large chain (Fragment), putative ftsH extracellular protease, putative RNA-binding (RRM/RBD/RNP motifs) family proteins, and dirigent proteins. Simultaneously, many proteins exhibit significant downregulation. These include gamma-tubulin complex component, putative pre-mRNA splicing factor, putative selenoprotein, Rdx type, putative RZZ complex, subunit Zw10, and putative golgin candidate 6. In 2 d seedling cotyledons raised in the presence of NaCl, 66 proteins exhibit a noteworthy regulation of their abundance ratio (Figure 8C). While proteins such as putative alpha/beta hydrolase fold protein, putative ABC2-like protein, ribulose bisphosphate carboxylase large chain (Fragment), putative ribulose bisphosphate carboxylase large chain, dirigent protein, and CAAX prenyl protease are upregulated significantly, a negative regulatory response has been noted in eukaryotic translation initiation factor 3 subunit D, putative pseudouridine synthase II, putative 30S ribosomal protein S1 protein, mitochondrial pyruvate carrier, and putative calcium-dependent protein kinase. The profile of 2 d old (−NaCl) and 2 d old (+NaCl) was evaluated in comparison to the proteome profile of OBMPs extracted from imbibed seeds.

Upon analysis of 2 d (+NaCl), using 2 d (−NaCl) as control, a significant regulation was noted in the OB MP profile (Figure 8C). Several proteins showed upregulation in their abundance ratio. These include putative lecithin:cholesterol/phospholipid:diacylglycerol acyltransferase, putative 11 kDa late embryogenesis abundant protein, putative RNA polymerase Rpb2, domain 2, DNA-directed RNA polymerase, subunit 2, putative pre-mRNA splicing factor, putative ABC2-like protein, putative selenoprotein, Rdx type, putative RZZ complex, subunit Zw10, putative golgin candidate 6, seed storage albumin 4, putative proton pump interactor 1, putative peroxin 22, putative seed maturation protein, putative dehydrin, and oleosin. However, some proteins showed downregulation. These include protein transport protein SEC23, putative 30S ribosomal protein S1 protein, putative insulinase (peptidase family M16) protein, putative histone H5, AT hook-like protein, ribosomal protein, eukaryotic translation initiation factor 3 subunit D, malate synthase, 6-phosphogluconate dehydrogenase, decarboxylating, putative MORN (membrane occupation and recognition nexus) repeat-containing protein, putative 14-3-3 protein 10, putative NADH-ubiquinone oxidoreductase, putative ADF/Cofilin/Destrin, and ADF-H/Gelsolin-like domain protein.

Post-translational modifications (PTMs) were noted in OBMPs extracted from imbibed seeds as well as control and stressed 2 d old seedling cotyledons of all three sunflower variants. While **19 OBMPs** exhibited significant PTMs to induce a tolerance response in the salt-tolerant variety—DRSH1, a total of **53 OBMPs** professed PTM regulation under salt stress in semi-tolerant variety—KBSH 53*.* The salt-sensitive variety, PSH 1962 revealed **33 OBMPs** to have demonstrated noteworthy PTMs at various positions. In all sunflower varieties, lipoxygenase, malate synthase, storage proteins—globulin, LEA, allene oxide synthase (AOS), legumin, class A and B and albumins (2S, 8, 1, 2, and 11S), and various members of heat shock proteins (HSPs) asseverate interesting modifications resulting due to post-translational modifications at various positions. Interestingly, semi-tolerant variety KBSH 53 revealed acetylation of the putative major intrinsic protein aquaporin-like protein at the *N* term. Additionally, putative calmodulin protein expression displayed methylation at positions K116 and R127 in the semi-tolerant variety, KBSH 53. Similarly, in the salt-sensitive (PSH1962) variety, the oleosin protein displayed acetylation at position K−117.

### Noteworthy regulation of OBMPs is evident in the seedling cotyledons in response to salt stress across the three sunflower variants

Based on the extensive research conducted on eco-physiological response of plants to salinity, oleosins have been identified as the most abundant membrane protein anchored to OBs and precariously absent from the vegetative tissues.^48^^,^^49^

***Oleosins*** (15−0 kDa) are highly conserved membrane proteins, with an idiosyncratic architecture that makes up to 79% of the OB’s surface.^47^ Well-adapted to withstand adverse environmental conditions, oleosins provide OBs with a significant interface for physiological activities.^1^ During seed germination, oleosins are gradually mobilized from OBs owing to the increasing activity of lipases and thiol proteases.^29^^,^^50^ In the present study, it has been determined through biochemical as well as and proteomic analyses that the abundance ratios of oleosins are highly reduced or undetectable in salt-tolerant, semi-salt-tolerant, and salt-sensitive varieties of sunflower, indicating their rapid mobilization. Small oleosin fragments are obtained from the salt-tolerant variety (DRSH1). The proteomic findings (present work) are in congruence with the OBMP mobilization pattern observed by SDS–PAGE analysis of biocarbonate-washed proteins (Figure 3; Table 5). Similarly, when the seedlings were raised in the presence of 120 mM NaCl, no shift in abundance ratios was observed in DRSH 1 (salt-tolerant variety). In semi-salt-tolerant and salt-sensitive varieties, however, an upregulation of oleosin abundance ratio confirms the delay in OB mobilization under salt stress in salt-sensitive variety (Table 6). Oleosins play a remarkable role by conserving TAG reservoirs in salt-sensitive seedlings under stress. Similar findings have been observed by SDS–PAGE analyses of bicarbonate-washed OBMPs, which support the proteomic findings (Figure 3). The correlation between salt tolerance and enhanced proteolytic degradation of OBMPs, such as oleosins, has been demonstrated earlier using sunflower variants in the author’s laboratory.^4^ Recent work on sheepgrass salt variants, tolerant and sensitive, has revealed a significant increase in the levels of LcOBAP, a form of oleosin expressed under salinity stress.^51^ In addition to the ubiquitous expression pattern of oleosins in OBMPs extracted from all three sunflower salt variants, several other OBMPs have exhibited noteworthy modulation owing to their differential salt-tolerance capacities. The OBMPs are discussed in depth in the text provided below.

**Table 5.** Differential regulation of oil body membrane proteins (OBMPs) in the cotyledons of seedlings raised in control conditions, in comparison to cotyledons from imbibed seeds.

S. No.	Protein (s)	Salt-tolerant variety (DRSH 1)	Salt-semi-tolerant variety (KBSH 53)	Salt-sensitive variety (PSH 1962)
1	Dirigent protein OS = *Helianthus annuus* OX = 4232 GN = DR206 PE = 3 SV = 1	Upregulated	NA	Upregulated
2	Seed storage albumin 4/2 OS = *Helianthus annuus* OX = 4232 GN = SESA4 PE = 2 SV = 1	Downregulated	Upregulated	Downregulated
3	Uncharacterized protein OS = *Helianthus annuus* OX = 4232 GN = HannXRQ_Chr17g0556691 PE = 4 SV = 1	Downregulated	NA	Downregulated
4	Malate synthase OS = *Helianthus annuus* OX = 4232 GN = MASY PE = 3 SV = 1	Upregulated	NA	Upregulated
5	Putative seed maturation protein OS = *Helianthus annuus* OX = 4232 GN = HannXRQ_Chr06g0170021 PE = 4 SV = 1	Downregulated	NA	Downregulated
6	Putative dehydrin OS = *Helianthus annuus* OX = 4232 GN = HannXRQ_Chr15g0482351 PE = 3 SV = 1	Downregulated	Downregulated	NA
7	Putative allene oxide synthase OS = *Helianthus annuus* OX = 4232 GN = C74A2 PE = 3 SV = 1	Upregulated	NA	Upregulated
8	Putative NAD(*P*)H dehydrogenase B2 OS = *Helianthus annuus* OX = 4232 GN = NDB2 PE = 4 SV = 1	Upregulated	NA	Downregulated
9	Putative leucine-rich repeat (LRR) family protein OS = *Helianthus annuus* OX = 4232 GN = HannXRQ_Chr13g0407051 PE = 4 SV = 1	NA	Downregulated	Upregulated
10	Putative legumin B OS = *Helianthus annuus* OX = 4232 GN = LEGB PE = 3 SV = 1	Downregulated	Upregulated	NA
11	Oleosin OS = *Helianthus annuus* OX = 4232 GN = OLEO6 PE = 3 SV = 1	Highly downregulated (oleosin fragment)	Downregulated	NA
12	Heat shock protein	Downregulated	Highly upregulated	Downregulated
	Types of HSP noted in the three variants –	***Putative chloroplast heat shock protein 70*** OS = *Helianthus annuus* OX = 4232 GN = HannXRQ_Chr03g0067941 PE = 3 SV = 1	***17.9 kDa class II heat shock protein*** OS = *Helianthus annuus* OX = 4232 GN = HSP17.9 PE = 2 SV = 1	***Putative 18.2 kDa class I heat shock protein*** OS = *Helianthus annuus* OX = 4232 GN = HSP12 PE = 3 SV = 1

**Table 6.** Differential regulation of oil body membrane proteins (OBMPs) upregulated, unregulated (NA), or downregulated in salt-stressed 2 d old seedling cotyledons (2 d 120), in comparison to cotyledons from imbibed seeds.

S. No.	Protein (s)	Salt-tolerant variety (DRSH 1)	Salt-semi-tolerant variety (KBSH 53)	Salt-sensitive variety (PSH 1962)
1	Ribulose bisphosphate carboxylase large chain (Fragment) OS = *Helianthus annuus* OX = 4232 GN = rbcL PE = 3 SV = 1	NA	Upregulated	Highly upregulated
2	Dirigent protein OS = *Helianthus annuus* OX = 4232 GN = DR206 PE = 3 SV = 1	Highly upregulated	NA	Highly upregulated
3	Putative leucine-rich repeat (LRR) family protein OS = *Helianthus annuus* OX = 4232 GN = HannXRQ_Chr13g0407051 PE = 4 SV = 1	NA	Highly upregulated	Upregulated
4	Putative calcium-dependent protein kinase OS = *Helianthus annuus* OX = 4232 GN = CDPK PE = 4 SV = 1	Highly upregulated	NA	Downregulated
5	Putative triacylglycerol lipase-like 1 OS = *Helianthus annuus* OX = 4232 GN = ATTLL1 PE = 4 SV = 1	NA	Downregulated	Upregulated
6	Putative allene oxide synthase OS = *Helianthus annuus* OX = 4232 GN = C74A2 PE = 3 SV = 1	NA	Highly downregulated	Upregulated
7	Putative 50S ribosomal protein L12 protein OS = *Helianthus annuus* OX = 4232 GN = RK12 PE = 3 SV = 1	Highly upregulated	NA	Upregulated
8	Putative import inner membrane translocase subunit Tim17/Tim22/Tim23 family protein OS = *Helianthus annuus* OX = 4232 GN = HannXRQ_Chr10ga0283991 PE = 4 SV = 1	NA	Upregulated	Upregulated
9	Putative translocase of the inner mitochondrial membrane OS = *Helianthus annuus* OX = 4232 GN = HannXRQ_Chr17g0534931 PE = 3 SV = 1	Highly upregulated	Highly upregulated	NA
10	Putative major intrinsic protein, Aquaporin protein OS = *Helianthus annuus* OX = 4232 GN = HannXRQ_Chr03g0086761 PE = 3 SV = 1	Highly upregulated (putative aquaporin PIP2−5)	Highly upregulated (aquaporin-like protein)	NA

Twelve OBMPs exhibit varying degrees of significant regulation across the three sunflower salt variants analyzed. Unique regulation is discerned in dirigent protein, which is upregulated in both salt-tolerant and -sensitive variety. Contrary to this, seed storage albumin 4/2 protein is downregulated in salt-tolerant and -sensitive variety and upregulated in the semi-tolerant variant. Putative dehydrin protein is significantly downregulated in both tolerant and semi-tolerant variants. Regulation of putative NAD (*P*) H dehydrogenase, putative leucine-rich repeat (LRR) family, putative legumin B, oleosin and heat shock protein shows a stark contrast in its expression pattern noted in tolerant, semi-tolerant, and sensitive sunflower variants (Table 5).

A significant differential regulation of the abundance ratios of 10 OBMPs is evident in the three varieties of sunflower in response to salt stress. Interesting upregulation of various proteins has been noted in salt-sensitive variety (PSH 1962). These proteins include ribulose bisphosphate carboxylase large chain (fragment), dirigent proteins, putative leucine-rich repeat (LRR) family protein, putative TAG lipase-like 1, allene oxidase synthase, 50S ribosomal protein L12 protein, and putative import inner membrane translocase subunit Tim17/22/23 family protein. Coherently, major proteins downregulated under stress conditions (Control: Imbibed seeds) include putative calcium-dependent protein kinase and putative intrinsic protein—aquaporin. Aquaporins like PIP5 and aquaporin-like proteins are highly upregulated and abundantly expressed in salt-tolerant and semi-tolerant sunflower variety, respectively (Table 6).

With reference to control seedling cotyledons, the OBMP profile of salt-stressed 2 d old seedling cotyledons highlights the expression patterns, regulation, and abundance ratios of eight unique proteins. Primarily, malate synthase and 50S ribosomal L12 protein exhibit downregulation in their abundant ratios as a vulnerability response to ameliorate salt stress by the salt-sensitive variety (PSH 1962). Additionally, proteins such as GDSL-like lipases, oleosins, and glutelin type B display are upregulated in response to salinity. Putative ABC transporter F and putative seipin family proteins demonstrate a unique rise in their abundance ratios and regulation in the salt-tolerant variety (Table 7).

**Table 7.** Differential regulation of oil body membrane proteins (OBMPs) found to be upregulated, unregulated (NA), or downregulated in salt-stressed 2 d old seedling cotyledons (2 d 120), in comparison to control seedling cotyledons.

S. No.	Protein	Salt-tolerant variety (DRSH 1)	Salt-semi-tolerant variety (KBSH 53)	Salt-sensitive variety (PSH 1962)
1	Malate synthase OS = *Helianthus annuus* OX = 4232 GN = MASY PE = 3 SV = 1	NA	Highly downregulated	Downregulated
2	Putative GDSL-like lipase/acylhydrolase superfamily protein OS = *Helianthus annuus* OX = 4232 GN = HannXRQ_Chr02g0039251 PE = 3 SV = 1	NA	Downregulated	Upregulated
3	Oleosin OS = *Helianthus annuus* OX = 4232 GN = OLEO PE = 3 SV = 1	NA	Upregulated	Upregulated
4	Putative 50S ribosomal protein L12 protein OS = *Helianthus annuus* OX = 4232 GN = RK12 PE = 3 SV = 1	Highly downregulated (Ribosomal L16)	Upregulated (L12)	Downregulated (L12)
5	Putative glutelin type-B 1 OS = *Helianthus annuus* OX = 4232 GN = GLUB1 PE = 3 SV = 1	NA	Slightly upregulated	Slightly upregulated
6	Uncharacterized protein OS = *Helianthus annuus* OX = 4232 GN = HannXRQ_Chr17g0556691 PE = 4 SV = 1	NA	Slightly upregulated	Upregulated
7	Putative ABC transporter F family member 2 OS = *Helianthus annuus* OX = 4232 GN = ABCF2 PE = 4 SV = 1	Highly upregulated	NA	NA
8	Putative seipin family OS = *Helianthus annuus* OX = 4232 GN = HannXRQ_Chr05g0141781 PE = 4 SV = 1	Highly upregulated	NA	NA
9	Putative calmodulin OS = *Helianthus annuus* OX = 4232 GN = CALM PE = 3 SV = 1	NA	Highly upregulated	Slightly downregulated

***Aquaporins (AQP)*** are multifunctional membrane proteins that regulate plant water relations across cellular biological membranes.^52^^,^^53^ Putative aquaporin members of the family tonoplast intrinsic proteins (TIPs) 1, 2, and 3 have earlier been reported as persistent constituents of oleosome membrane on the OBMPs extracted from *Arabidopsis thaliana* and *Arachis hypogaea.*^47^^,^^54^ Furthermore, aquaporins have been reported as significant members of OBMPs from various other plant species, such as *Helianthus annuus*, *Gevuina avellana*, *Madia sativa,* and *Triadica sebifera.*^55-57^ OBs exhibit essential physical interaction with several subcellular organelles, including ER, mitochondria, vacuoles, peroxisomes (glyoxysomes in seedlings), plasma membrane, and plasmodesmata, for their biogenesis, delivery of biomolecules and regulation of storage lipids.^7^^,^^58^^,^^59^ Recently from author’s laboratory, a novel crosstalk has been reported between polyamine metabolism and mobilization of OBs in sunflower seedlings. This revealed the role of aquaporins (TIPs and PIPs) in OB mobilization under normal as well as saline growth conditions.^55^ Similarly, various isoforms of TIPs have been reported in response to salinity in ice plant (*Mesembryanthemum crystallinum*).^53^^,^^60^ Our work depicts the regulation of putative Aquaporin PIP2 and PIP5 in the cotyledons of 2 d old, salt-tolerant sunflower varieties (DRSH1) under salinity stress. Similarly, in semi-salt-tolerant proteins, ***aquaporin-like proteins*** **(PIP2 and PIP5)** exhibit high accumulation under stressful environmental conditions (Table 6). In congruence with our findings on the effect of salt stress, overexpression of TaNIP (an isoform of aquaporin) of wheat in transgenic Arabidopsis seedlings shows significant upregulation, imparting salt tolerance.^61^ TIPs and PIPs whose levels are induced in leaves under salinity stress may probably be associated with water influx in leaves.^52^^,^^62^ Plasma membrane intrinsic proteins (PIPs) majorly drive the flux of plant water interactions. PIP2 members exhibit much more effective water transport than the PIP1 family owing to their distinct molecular structure compared to the PIP1 isoforms.^63^ In both salt-tolerant and semi-salt-tolerant sunflower seedlings, a significantly increased accumulation of aquaporin proteins observed on the monolayer of the oleosome’s membrane is an interesting finding that has established concretely the role of aquaporins in building a stronger tolerance mechanism in sunflower. Similar observations pertaining to the overexpression of aquaporins have been linked to improved phenotypes in various economically important crop species, such as wheat, rice, soybean, tomato, populus, and banana.^52^^,^^64-69^ However, in salt-sensitive sunflower seedling (PSH1962), aquaporin levels on the OB membrane remain unregulated under a saline environment (Table 6). As indicated by recent data, salinity and drought stress induce maximum alterations in expression levels of aquaporins, especially PIPs and TIPs.^70^^,^^71^ Identified as elemental gatekeepers of the cell, these findings are crucial for establishing the potential of aquaporins as paramount regulators of salt tolerance in sunflower.

An interesting physiological expression is the post-translational modification of aquaporins via phosphorylation of serine residues that results in closing of these channels to regulate water movement under stressful abiotic conditions such as salinity and drought.^72^ This brings us to another formidable player noted in our study, responsible for imparting salt-tolerance—CPKs: calcium-dependent protein kinases.

***Calcium-dependent protein kinases (CPKs)*** are remarkable and versatile calcium-binding proteins which sense and regulate cytosolic calcium concentrations for directing an adaptive response under varied environmental conditions.^73^ CPKs have been categorized as positive regulators of saline stress in a small number of plants such as rice, cotton, Arabidopsis, and wheat. Their cellular and subcellular containment to membranes under stress indicates its keynote role in signal transduction.^74^^,^^75^ Plants of *Oryza sativa* raised under sodic conditions require CPK5 and CPK13 for complete activation of mitogen-activated proteins kinases (MAPKs), which act against amelioration of stress levels due to elevated levels of sodium ions.^76^ Localization studies on CPKs in plants have revealed their widespread occurrence in different plant tissues such as leaves, meristems, roots, and flowers of various plant species, such as wheat, rice, tomato, melon, maize, poplar, grapevine, and many more. A cascade of Ca^2+^-triggered responses involving a chain of CPKs is elucidated as regulators of the osmotic stress response in plants.^77^^,^^78^ In the current investigations, the localization of CPKs on the membrane of oleosomes isolated from a salt-tolerant variety (DRSH1) grown under salt stress indicates its unique perseverance in stimulating salt-tolerance. In contrast, this protein exhibits downregulation in salt-sensitive (PSH 1962) seedling cotyledons grown in salt-stressed conditions (Table 6, S. No. 4).

In Arabidopsis mutant, *cpk27−1*, inactivation of CPK 27 results in irreversible salt sensitivity, with plantlets incapable of extruding noxious ions and high retention of radicals at germination and post-germination levels. Similarly, in *atcpk1* mutants, elevated accumulation of H_2_O_2_ and malondialdehyde (MDA) and a decrease in proline accumulation are observed.^79^^,^^80^ Although the activation process of CPKs is unclear, it is postulated based on recent findings by *Qingzhong Li* & group that osmo-regulated phosphorylation of CPKs such as 3/4/6/11 and 27 triggers sensitization to induce salt tolerance.^77^ More conclusive works need to be carried out to establish the role of calcium and related CPKs in oleosomes and in ameliorating salt tolerance in variants of sunflower.

Another membrane proteins from OBs—***SEIPIN*** protein—has been noted in its highly upregulated form in the salt-tolerant variety, DRSH1 under extracted salt-tolerant of sunflower (Table 7). Seipin is a highly conserved oligomeric ER transmembranal protein that acts as a protective gatekeeper of lipid flux at the nexus of the endoplasmic reticulum–oil body.^81^^,^^82^ Interestingly, Seipin proteins are membranal markers that primes the ER sites for OB biogenesis.^83^^,^^84^ Significant modulations in the expression of OB proteins are noted in the seeds of salt-tolerant and salt-sensitive variants of sheepgrass upon exogenous application of 300 mM NaCl.^51^ In the present work, a unique surge in putative seipin proteins is noted in the salt-tolerant variety of sunflower under salt stress (Table 7). Understanding the exact mechanism of function of Seipins is still underway. However, proteomic evidence has confirmed its role as a territorial marker for inducing biogenesis in the tolerant sunflower variety DRSH1 under salinity stress. More confirmatory work is needed to further decipher its role in stress mitigations.

***Dirigent (DIR) protein*** associated with peroxidases modulates lignification of plant cell walls during cell growth and germination, and under stressful environmental conditions. Expression of DIR-*like* genes is modulated in response to water stress, abscisic acid, and cold conditions in *Arabidopsis.*^85-87^ A specific DIR, the *At*DIR3 gene, has motif matrix transcription factors specific to seed growth and often disposes of independent regulation during growth.^86^^,^^88^ In *Pepper capsica* and *Capsicum annuum* L., the upregulation of several homologs of DIR genes, such as *CaDIR2, CaDIR3, CaDIR6, CaDIR11, CaDIR22,* and so on, has been mapped to reveal the constitution of the DIR gene in lignin biosynthesis.^89^ In the current investigations, dirigent protein has shown noteworthy upregulation in 2 d old control OBMPs as compared to the imbibed seeds, irrespective of their differential salt-tolerant abilities (Table 5). In congruence with these observations, genome analysis of the *Arabidopsis thaliana* mutant *atdp1* revealed the crucial mantle of *AtDIR1/AtDIR12* as a precursor for the biosynthesis of neolignins in the leaves and for their role in increasing seed coat permeability, thus affecting the seed germination response.^90^ Similarly, in the present work, OBMPs from stressed seedling cotyledons (compared to imbibed seeds) exhibit a more prominent upregulation in both salt-tolerant and salt-sensitive varieties (Table 6). This indicates the importance of dirigent protein in ensuring proper seed germination and growth under conditions of stress. In seedlings raised under 120 mM NaCl (present work), pronouncement of DIR proteins highlights the ability of this protein to ameliorate salt stress to facilitate growth and development at the seedling level.

***Plant dehydrins DNH**,* commonly known as Group II late embryogenesis abundant (LEA) proteins, are disordered proteins with hydrophilic domains.^91^ In the present study, putative dehydrins have been observed to be downregulated in the cotyledons of 2 d old seedling cotyledons of both tolerant and semi-salt-tolerant varieties grown under control conditions (Table 5). However, no significant regulatory response was evident in the salt-sensitive variety. These observations can be attributed to the possible role of dehydrins as essential regulators of seed maturation and in managing abiotic stress tolerance. They are known to protect membrane integrity as well as protein and DNA conformations. By establishing cross talks with MAP kinases, ABA, and Ca^2+^ signal transduction, plant dehydrins are abundantly located in mature seeds in subcellular locations wherein they enable the mature seeds to maintain a state of dormancy.^91^^,^^92^ Thus, in the foregoing work, downregulation of DNH levels in tolerant and semi-tolerant sunflower variants is a tentative indicator of seed receptiveness for growth and development under stress conditions.

***Pyridine nucleotide [NAD(P)H] dehydrogenases**,* maintain the cellular homeostasis and survival of plants by regulating nucleotide metabolism. Thus, they play diverse roles in seed germination, pollen tube growth, stomatal opening, plant metabolism (photosynthesis, photorespiration, respiration, and glycolysis), signal transduction, gene expression, and biofortification.^93-96^ Notably, salt-tolerant plants synthesize purines at a much faster rate than salt-sensitive plants do, especially under conditions of salinity stress, enabling the plants to conduct normal physiological processes efficiently.^93^ In the present work, the concentration of NAD(*P*) dehydrogenase B2 (NDB2) displayed intriguing regulation in the OBMP pool of 2 d old seedling cotyledons. While in the salt-tolerant variety (DRSH1), a significant positive regulation of the enzyme’s abundance was noted, in case of the salt-sensitive variant (PSH 1962), it exhibited a negative regulation in its abundance ratio (Table 5). A major function of the NAD(*P*) dehydrogenase B2 family in *Arabidopsis thaliana* has been identified as proteins associated with alternative oxidase (AOX), where they enable the redox status of the cells to develop abiotic stress-tolerant phenotypes.^97^^,^^98^ The foregoing work clearly depicts the abundance of NDB2 [NAD(*P*) dehydrogenase B2] in OBMPs isolated from salt-tolerant sunflower variants and its subsequent absence from salt-sensitive variety (PSH 1962). These observations are indicative of the role of dehydrogenase class B2 in the transmittance of salt tolerance via the regulation of oil bodies.

***Leucine-rich repeat (LRR)*** and its associated receptor-like kinases (RLKs) are transmembrane proteins that function as cornerstone sensors and constitute the largest group of receptor-like kinases in plants for developmental and defense-related responses.^99^^,^^100^ Highly conserved sequences and residues comprise an LRR motif which rapidly perceives ligands as receptors.^101^ Crucial for the identification of lacunas in either development or defense-related mechanisms, LRR proteins recognize extracellular signals of distress and coordinate plant growth and immunity in a timely fashion. Identified as an immunity-lever, LRRs are critical for ensuring normal growth of the plant by pronouncement of SAM and RAM development.^100^^,^^102^ Similarly, associated with the cell membrane and wall of *A. thaliana*, leucine-rich repeat extension proteins (LRXs), namely LRX3, LRX4, and LRX5, have been demonstrated to be critical input in the maintenance of plant stress tolerance at the seedling stage.^103^ Around 233 genes of LRR-RLK have recently been constituted for roles in plant growth and stress regulation in *Liriodendron chinense*.^104^ In the present work, no significant regulation of LRR proteins was detected in the OBMPs of control seedlings of the salt-tolerant sunflower variety (DRSH1) (Table 5). However, under salt stress, the seedling cotyledons of semi-tolerant (KBSH 53) and salt-sensitive (PSH 1962) varieties exhibit a significantly high upregulation of LRRs and associated RLKs (Table 6). Interestingly, in the salt-sensitive variety, increased levels of LRR proteins are observed in both control and salt-stressed seedling cotyledons (Tables 5 and 6).

Oxylipins are a superfamily of oxygenated fatty acid derivates that are subject to metabolism by a group of enzymes called ***allene oxide synthases AOS***^105^ AOSs are pivotal regulatory enzymatic groups associated with the biosynthesis of various non-volatile oxylipins, such as jasmonic acid (JA), which has established roles in plant development and in managing stressful environmental signals.^105-107^ Stress-mediated initiation/biosynthesis of AOSs has been documented in various plants, such as rice, barley, tomato, Arabidopsis, and potato. In rice plants, AOS levels increase significantly following infection or attack by any herbivore, resulting in an intensified defense response.^106^ AOS-derived compounds have recently been identified from the roots of young wheat and rice seedlings and are collectively termed “graminoxins.”^108^ Interestingly, stress-induced, cyclic AOS products, termed “death acids,” have also been discovered in maize.^109^ In the present work on sunflower, the abundance ratio and levels of AOS are enhanced both in control and salt-stressed seedlings of the salt-sensitive variety (PSH 1962). AOS levels also undergo upregulation under salt stress in the semi-tolerant variety (KBSH 53) (Tables 5 and 6). These observations are in congruence with the above-cited literature, stipulating a remarkable role of AOS in triggering a defense mechanism in the susceptible variety of sunflower under salinity stress.

***ATP-binding cassette (ABC) proteins*** are transmembrane entities that regulate plant growth and ameliorate well-curated responses against abiotic stress.^110^ All eight major subfamilies of ABC transporters—from ABCA to ABCG and ABCI—play a variety of roles and responses in plants.^111^ Some ABC transporters are associated with plastid lipid biosynthesis and the regulation of seed germination.^111-113^ However, not much work has been carried out on stress-related ABC transporters. Recently, in *Zea mays*, the ABC gene *ZmMPRA6* has been analyzed for its ability to impart tolerance against cold and salinity stress.^114^ In rice plants, the ABC transporter G subfamily has been reported to impart salt tolerance.^115^ In the salt-tolerant variety of sunflower (DRSH1), a unique and significant upregulation in the expression of *HaABCF2* transporters is evident in response to salt stress (Table 7). Recent investigations of the ABC gene family in the *Pyrus bretchneideri* (Pear) genome revealed the expression of 177 ABC transporter genes. Among the identified subfamilies, eight genes belong to the ABC transporter F-subfamily. Interestingly, the expression profiles of *PbrABCF1* and *4* are upregulated in response to salt and drought stress, whereas the expression of *PbrABCF2* is initially downregulated.^116^ Similarly, in rice, salt stress results in the downregulation of ABC transporters belonging to *Os*ABCF5.^117^ In *Arabidopsis thaliana, At*ABCF3 is involved in the upregulation of aquaporins, which, in turn, modulates the levels of hydrogen peroxide in roots.^111^^,^^118^ The current work in sunflower varieties revealed the unique expression of the stress-related transporter *HaABCF2* from the oleosome membrane of salt-tolerant DRSH 1 under salinity stress. In semi-tolerant and salt-sensitive seedlings, ABCF2 transporters were not detected. This finding provides evidence for a direct association of ABC transporters in imparting salt tolerance to the salt-tolerant variety, DRSH 1.

***Malate synthase*****,** a crucial enzyme of the glyoxylate cycle, undergoes a prompt surge after imbibition and decreases during post-germinative development. The levels of MS are strongly associated with lipid breakdown in Arabidopsis, cucumbers, and orchids.^119-121^ Using malate synthase, insertional mutation investigation has been carried out to decipher its mode of action and precise impact on salt-tolerance abilities of a mutant line of Arabidopsis.^122^ In different sunflower varieties under present investigation, this enzyme is upregulated in both 2 d old salt-tolerant (DRSH1) and salt-sensitive (PSH 1962) seedling cotyledons (unstressed), showing coherence with the cited literature (Table 5). The levels of malate synthase remain unaffected in salt-tolerant seedling cotyledons. However, in semi-tolerant and salt-sensitive sunflower variants, *Ha*MS levels decline drastically indicating the correlation between tolerance receptivity and metabolic responses (Table 7). It is thus becoming evident that for surviving in the harsh environmental stress, animals, plants, and microbes have evolved malate synthase as one of the regulatory enzymes to maintain plant growth and metabolism under oxidative stressful conditions.^123^

### Post-translational modifications noted in different sunflower varieties under salinity stress

As discussed in the above-mentioned results, significant PTMs have been noted in several OBMPs. However, a few that stood out include aquaporins and calmodulin (semi-tolerant variety) and oleosin (salt-sensitive variety). In the semi-tolerant variety, KBSH 53, acetylation and methylation of aquaporins and calmodulin have been noted, respectively. Similarly, in the salt-sensitive variety (PSH1962), acetylation of oleosin protein was observed. To support the observations made in sunflower, a comparison can be drawn to the profile of co- and post-translational modifications in sesame seeds via MS analyses (proteomics) which revealed their importance in enhancing the structural stability of OBs via limiting ubiquitination-mediated degradation. Commonly recognized post-translational modifications of oleosins, caleosins, and steroleosins of OB membrane include acetylation of methionine and deamination of glutamine residues, which result in the introduction of a negative surface charge, consequently leading to the reinforcement of structural stability in the OBs.^2^^,^^124^^,^^125^ Thus, it can be concluded that the role of co- and post-translational modifications is crucial and elemental for ensuring the structural stability and functional integrity of OBs in plants.

## Conclusion and perspective

The current investigations demonstrate an intricate interplay of various proteins that are responsible for imparting salt tolerance to sunflower seedlings. Various varieties respond differently to NaCl. Numerous proteins have been identified as the components of the oil body membranes. Oleosins function to block the fusion of oil bodies, suggesting possible interactions among oleosomes and other organelles, such as endoplasmic reticulum, protein bodies, and mitochondria. Some proteins, such as enolase, alcohol dehydrogenase, malate dehydrogenase, aldolase, phosphoglycerate kinase, and glyceraldehyde-3-phosphate dehydrogenase, are cytosolic proteins, and they might possibly be appearing as contaminants during the washing of OBMPs prior to LC‒MS/MS analysis. In fact, some of the hydrophobic cytosolic proteins are likely to adhere to oil bodies, thereby making their separation difficult during the OB washing procedure even with stringent procedures using urea. Bioengineering of lipid metabolism and synthetic biology stratifications are the ongoing intensive research domains wherein high yielding TAG lines of oilseed crops can play a major role in the development of sustainable lines, considering the surge of consequences due to climate change. The mechanisms of salt tolerance can be better deciphered to provide a knowledge pool to cater to physiologists, biotechnologists, and agricultural biologists for constructing tolerance mechanisms in major cultivated crops. A recent concept, known as “push–pull–protect,” has resulted in the expansion of TAG accumulation through engineered co-production of plant seed proteins that increase the flux of TAGs in vegetative tissues and seeds. Artificial oleosins have been developed in some laboratories to profess TAG packaging. This is a pathbreaking research that could alter the future of oilseed crop production. The impact of salt stress on the crop production of oil-yielding plants can further be managed by introducing techniques such as “push–pull–protect” and “systems biology-based artificial oleosins” in potential salt-tolerant and salt-sensitive varieties, thereby paving way for the agricultural sector to bloom.

## Authors' contributions

SCB conceptualized the research plan. MG undertook the experimental part. The manuscript was jointly written by both the authors. Illustrations were prepared by MG.

## Data Availability

All the supplementary data for the present work is available from the corresponding author.
